# Associations between psychological distress and financial hardship for patients with cancer receiving care in Alabama

**DOI:** 10.1007/s11764-026-02012-6

**Published:** 2026-04-01

**Authors:** Courtney P. Williams, Sandra Olisakwe, Chloe J. Taub, Margaret I. Liang, Gabrielle B. Rocque, Maria Pisu

**Affiliations:** 1 Division of General Internal Medicine & Population Science, University of Alabama at Birmingham, 1720 2 Ave South, BDB 852, Birmingham, AL 35294 USA; 2 O’Neal Comprehensive Cancer Center, Birmingham, AL USA; 3 Division of Hematology & Oncology, University of Alabama at Birmingham, Birmingham, AL USA; 4 Division of Gynecologic Oncology, Department of Obstetrics & Gynecology, Cedars-Sinai Medical Center, Los Angeles, CA USA

**Keywords:** Financial hardship, Financial toxicity, Distress, Screening, Patient-reported outcomes, Southern US, Alabama

## Abstract

**Purpose:**

To optimize integration of appropriate referral pathways to adequate resources, a better understanding of the relationship between psychological distress and financial hardship for patients with cancer in Alabama is needed.

**Methods:**

This cross-sectional study used standard-of-care collected, electronic, patient-reported outcome data from patients initiating medical oncology treatment. Psychological distress was measured using the NCCN Distress Thermometer. The FACIT-COmprehensive Score for financial Toxicity (COST) was used to measure cancer-related financial hardship. Associations between distress and financial hardship, domains of financial difficulty and distress, and factors associated with financial hardship and distress both alone and co-occurring were examined.

**Results:**

Of 4637 patients with screening data, almost one-third (31%) reported both moderate/severe financial hardship and moderate/severe distress. Patients with financial hardship had nearly three times the odds of distress compared to those without financial hardship (aOR 2.99, 95% CI 2.61–3.44). Patients with co-occurring financial hardship and distress more often reported employment and disability issues and difficulty affording utilities and basic needs than those with financial hardship only. Factors associated with reporting both financial hardship and psychological distress included patients who were younger, less educated, unmarried, female, non-White, and resided in highly disadvantaged neighborhoods.

**Conclusion:**

Both psychological distress and financial hardship were common and co-occurring for Alabama patients with cancer, with almost one-third of patients experiencing both financial hardship and distress.

**Implications for Cancer Survivors:**

Integration of financial hardship and psychological distress screening, with referrals to interdisciplinary interventions such as psycho-oncology services, could address both financial and psychological needs of patients with cancer.

**Supplementary Information:**

The online version contains supplementary material available at https://doi.org/10.1007/s11764-026-02012-6.

## Background

Distress, defined as the biopsychosocial feelings of pain or suffering, is a common issue in oncology, affecting 22–58% of patients with cancer physically, psychologically, emotionally, socially, and spiritually [1, 2]. Patients with cancer experiencing distress are at increased risk of adverse outcomes compared to those not experiencing distress, including poor quality of life, treatment non-adherence, increased physical symptoms, and higher mortality rates [3]. Because of these identified adverse outcomes, the American College of Surgeons’ Commission on Cancer has mandated routine, standard-of-care distress screening of patients receiving care at their accredited cancer centers since 2015 [4, 5]. This routine distress screening, most often completed at cancer treatment initiation, allows early identification and referral to supportive services to address sources of distress and, thus, improve well-being and cancer outcomes. However, pragmatic implementation of distress screening has been inconsistent, with estimates of 47–73% of eligible patients being screened, and of those identified as distressed, only 50–63% having documentation of referrals to psychosocial care [6]. These inconsistencies in implementation may be due in part to a lack of understanding of the key drivers of cancer-related distress and of the personalized referral pathways that may be necessary to address them.

Cancer-related distress is associated with a multitude of detectable and modifiable factors, potentially including cancer-related financial hardship, defined as the negative financial consequences of cancer care [7, 8]. Such financial hardship often results in adverse outcomes in the contexts of (1) material conditions, e.g., trading off paying for food to pay medical bills, (2) behavioral modifications, e.g., delaying/foregoing care due to costs, and (3) psychological consequences, e.g., increased distress [9]. As psychological distress represents a key domain of financial hardship, integrated approaches to screening may best adequately identify both distress and financial hardship [10]. However, it is unknown how often distress and financial hardship co-occur in patients with cancer. Furthermore, to understand how to optimize integration of appropriate referral pathways to adequate resources and successfully address cancer-related distress, a better understanding of the patients at highest risk for both psychological distress and financial hardship, as well as the types of financial difficulties patients with co-occurrence commonly experience, is needed.

A unique opportunity to better define the relationship between psychological distress and financial hardship exists at the University of Alabama at Birmingham O’Neal Comprehensive Cancer Center, where patients initiating medical oncology treatment are screened for both psychological distress and financial hardship as part of standard of care [10–12]. This study is of particular importance for patients receiving cancer care in the Deep South state of Alabama, and similar settings, where factors such as high poverty rates and logistical barriers to accessing cancer care (un/underinsurance, long travel distances) may increase risk of both psychological distress and financial hardship [13–16]. Therefore, this study assesses the prevalence of, associations between, and risk factors for financial hardship and distress for patients receiving cancer care in the state of Alabama.

## Methods

### Study design and setting

This exploratory cross-sectional study used standard-of-care collected, electronic, patient-reported outcome (ePRO) survey data from patients initiating medical oncology treatment at the University of Alabama at Birmingham (UAB) between October 2020 and November 2024 [17]. Surveys are administered at cancer treatment initiation by trained lay navigators either in person or by phone. Data from the ePRO surveys are embedded into the electronic medical record (EMR). Adults aged ≥ 18 years old with valid responses for the financial hardship and distress screening measures were included. This study was approved by the UAB Institutional Review Board (IRB-300007406).

### Primary outcomes: psychological distress and financial hardship

#### Psychological distress

Psychological distress was measured using the NCCN Distress Thermometer, a single-item visual analog scale ranging from 0 (no distress) to 10 (extreme distress) [18]. A score of 4 or greater is the recommended clinical threshold indicating moderate-to-severe distress and prompting psychosocial support referral [19]. Distress scores were categorized as none/mild (< 4) versus moderate/severe (≥ 4) distress.

#### Financial hardship

The FACIT-COmprehensive Score for financial Toxicity (COST) was used to measure cancer-related financial hardship [20, 21]. The scale includes 11 items scored from 0 to 44, with lower scores indicating greater financial hardship. For this analysis, financial hardship was defined as a COST score < 26, consistent with established thresholds for moderate-to-severe financial toxicity [11, 22, 23]. To provide additional context during the same screening, patients also reported whether they experienced financial difficulty across 10 domains, including costs of care (medications, supplies, upfront medical payments, insurance/medical bills), treatment logistics (transportation, lodging), basic needs (food/shelter, utilities), employment/disability issues, and child/elder care. Responses were dichotomized as no versus any financial difficulty [11].

#### Co-occurrence

An additional variable indicating co-occurrence of psychological distress and financial hardship was created via categorization into four mutually exclusive groups, including screening positive for (1) both moderate/severe psychological distress and moderate/severe financial hardship, (2) moderate/severe psychological distress only, (3) moderate/severe financial hardship only, or (4) neither.

### Covariables

Sociodemographic and clinical characteristics were abstracted from the EMR and survey platform at the time of treatment initiation, including age, sex (female, male), race (White, non-White), marital status (married, other), education level (high school or less, some college, bachelor’s degree or higher), patient home address, cancer type (breast, gastrointestinal, gynecologic, other), and cancer stage (early, advanced). Patient home address was used to classify residence as rural or urban according to the USDA Rural–Urban Commuting Area codes [24]. Rural residence includes patients living only in a non-urban core area, while urban residence includes patients living in both metropolitan and micropolitan central and outlying areas. Patient address was also used to classify those living in the least (percentile of 1–84) or most (percentile of 85–100) deprived neighborhoods according to the Area Deprivation Index [25, 26]. To minimize exclusions and retain full cohort size, we created missing-indicator categories for each covariate.

### Statistical analysis

Patient characteristics were summarized overall and by the four-level variable representing co-occurring financial hardship and psychological distress. Continuous variables were reported as medians with interquartile ranges (IQR) and categorical variables as frequencies and percentages.

#### Associations between distress and financial hardship

We first examined associations between distress and financial hardship descriptively using a calculation of the Pearson correlation coefficient (r). We then ran three covariable-adjusted models estimating (1) the linear relationship between distress and financial hardship using beta coefficients (β) and corresponding 95% confidence intervals (CIs) from a linear regression model, (2) difference in distress scores for patients with moderate/severe financial hardship (COST scores < 26) vs. no/mild financial hardship (COST scores ≥ 26) using β and 95% CIs from a linear regression model, and (3) odds of moderate/severe distress (NCCN Distress Thermometer score ≥ 4) vs. no/mild distress (NCCN Distress Thermometer score < 4) for patients with moderate/severe vs. no/mild financial hardship using adjusted Odds Ratios (aORs) and 95% CIs from a logistic regression model.

#### Financial difficulty domains and distress

For each of the ten financial difficulty domains, we calculated the proportion of patients reporting any difficulty and displayed these proportions both overall and by psychological distress (no/mild, moderate/severe).

#### Factors associated with financial hardship and/or distress

To identify medical and sociodemographic factors associated with each financial hardship and distress co-occurrence group, we fit a multinomial logistic regression model estimating odds of financial hardship only, distress only, or both financial hardship and distress vs. neither using aOR and 95% CIs. All statistical tests were two-sided with *α* = 0.05. Multicollinearity of model covariables was assessed using the variance inflation factor, with none identified. Analyses were conducted using SAS version 9.4 (SAS Institute Inc., Cary, NC).

## Results

### Study sample and characteristics

A total of 4637 patients completing ePRO surveys between October 2020 and November 2024 were included in this analysis, and 5162 patients were excluded due to non-reported financial hardship and/or distress screening measures (Supplemental Table 1). Included and excluded patients had similar distributions of sociodemographic and clinical characteristics. With a median age of 65 (IQR 53–73), our study sample was 65% female, 23% non-White, 22% with a high school education or less, 19% living in a highly disadvantaged neighborhood, 17% residing rurally, and 21% with an advanced stage cancer diagnosis. Almost one-third (31%) of our sample reported both moderate/severe financial hardship and moderate/severe distress, one-fourth (26%) reported moderate/severe distress only, 11% reported moderate/severe financial hardship only, and 32% reported neither (Table 1).

**Table 1:** Patient sociodemographic and clinical characteristics by reported financial hardship and distress (*N* = 4637)

	**Overall (*N* = 4637)**	**Both (*n* = 1433)**	**Distress only (*n* = 1230)**	**Financial hardship only (*n* = 500)**	**Neither (*n* = 1474)**
	***n* (%)**	***n* (%)**	***n* (%)**	***n* (%)**	***n* (%)**
**Age, median (IQR)**	65 (53–73)	58 (48–67)	67 (56–74)	61 (50–69)	69 (60–76)
**Sex**					
Female	3037 (65)	987 (69)	844 (69)	307 (61)	899 (61)
Male	1600 (35)	446 (31)	386 (31)	193 (39)	575 (39)
**Race**					
White	3163 (68)	873 (61)	907 (74)	301 (60)	1082 (73)
Non-White^a^	1080 (23)	413 (29)	216 (17)	154 (31)	297 (20)
Unknown	394 (9)	147 (10)	107 (9)	45 (9)	95 (7)
**Marital status**					
Married	2598 (56)	657 (46)	800 (65)	243 (49)	898 (61)
Other^b^	1846 (40)	703 (49)	387 (31)	243 (49)	513 (35)
Unknown	193 (4)	73 (5)	43 (4)	14 (2)	63 (4)
**Education**					
High school education/less	998 (22)	434 (30)	234 (19)	127 (25)	203 (14)
Some college^c^	1033 (22)	394 (28)	285 (23)	124 (25)	230 (16)
Bachelors’/higher	1409 (30)	355 (25)	488 (40)	130 (26)	436 (30)
Unknown	1197 (26)	250 (17)	223 (18)	119 (24)	605 (40)
**Neighborhood disadvantage**					
Least disadvantaged	2600 (56)	699 (49)	740 (60)	261 (52)	900 (61)
Most disadvantaged^d^	884 (19)	332 (23)	179 (15)	127 (26)	246 (17)
Unknown	1153 (25)	402 (28)	311 (25)	112 (22)	328 (22)
**Residence**					
Rural	773 (17)	261 (18)	177 (14)	89 (18)	246 (16)
Urban	2744 (59)	783 (55)	749 (61)	304 (61)	908 (62)
Unknown	1120 (24)	389 (27)	304 (25)	107 (21)	320 (22)
**Cancer type**					
Breast	1076 (23)	339 (24)	361 (29)	110 (22)	266 (18)
Gastro-intestinal	466 (10)	166 (12)	99 (8)	51 (10)	150 (10)
Gynecological	397 (9)	102 (7)	104 (9)	37 (8)	154 (11)
Other	2175 (47)	633 (44)	534 (43)	255 (51)	753 (51)
Unknown	523 (11)	193 (13)	132 (11)	47 (9)	151 (10)
**Cancer stage**					
Early	1060 (23)	286 (20)	340 (28)	99 (20)	335 (23)
Advanced^e^	982 (21)	274 (19)	213 (17)	134 (27)	361 (24)
Unknown	2595 (56)	873 (61)	677 (55)	267 (53)	778 (53)

^a^Black/African American, Hispanic/Latinx, Asian, American Indian/Alaskan Native, Multiple races

^b^Divorced, separated, widowed, single

^c^Associate’s or junior college degree

^d^Area Deprivation Index percentile of 85–100

^e^Stage III/IV

### Associations between distress and financial hardship

Descriptive models demonstrated a moderate inverse correlation between distress and financial hardship scores as measured by the COST instrument, indicating financial hardship and distress increase simultaneously (*r *= –0.37, *p* < 0.001). After adjustment for sociodemographic and clinical characteristics, adjusted models estimated that with every 10-point decrease in financial hardship as measured by COST, NCCN Distress Thermometer scores also increased by 1 point (*β* = 0.9, 95% CI 0.8–0.9; Table 2). Additionally, patients experiencing financial hardship had distress scores 1.7 points higher than those with no/mild financial hardship (95% CI 1.5–1.8). Furthermore, patients with moderate/severe financial hardship had nearly three times the odds of moderate/severe distress compared to those with no/mild financial hardship (aOR 2.99, 95% CI 2.61–3.44).

**Table 2:** Adjusted models estimating associations between measures of psychological distress and financial hardship (*N* = 4637)

	Distress (continuous NCCN Distress Thermometer score)	Distress (continuous NCCN Distress Thermometer score)	Moderate/severe distress (NCCN Distress Thermometer score 4–10 vs. 1–4)
Financial hardship (10-point increase in COST)	*β* = -0.9 95% CI -0.8– -0.9	–	–
Moderate/severe financial hardship (COST < 26 vs. ≥ 26)	–	*β* = 1.7 95% CI 1.5–1.8	aOR 2.99 95% CI 2.61–3.44

All models adjusted for age, race, sex, education, marital status, residence, neighborhood deprivation, cancer type, and cancer stage

### Domain-specific financial difficulty and distress

**Fig. 1:**
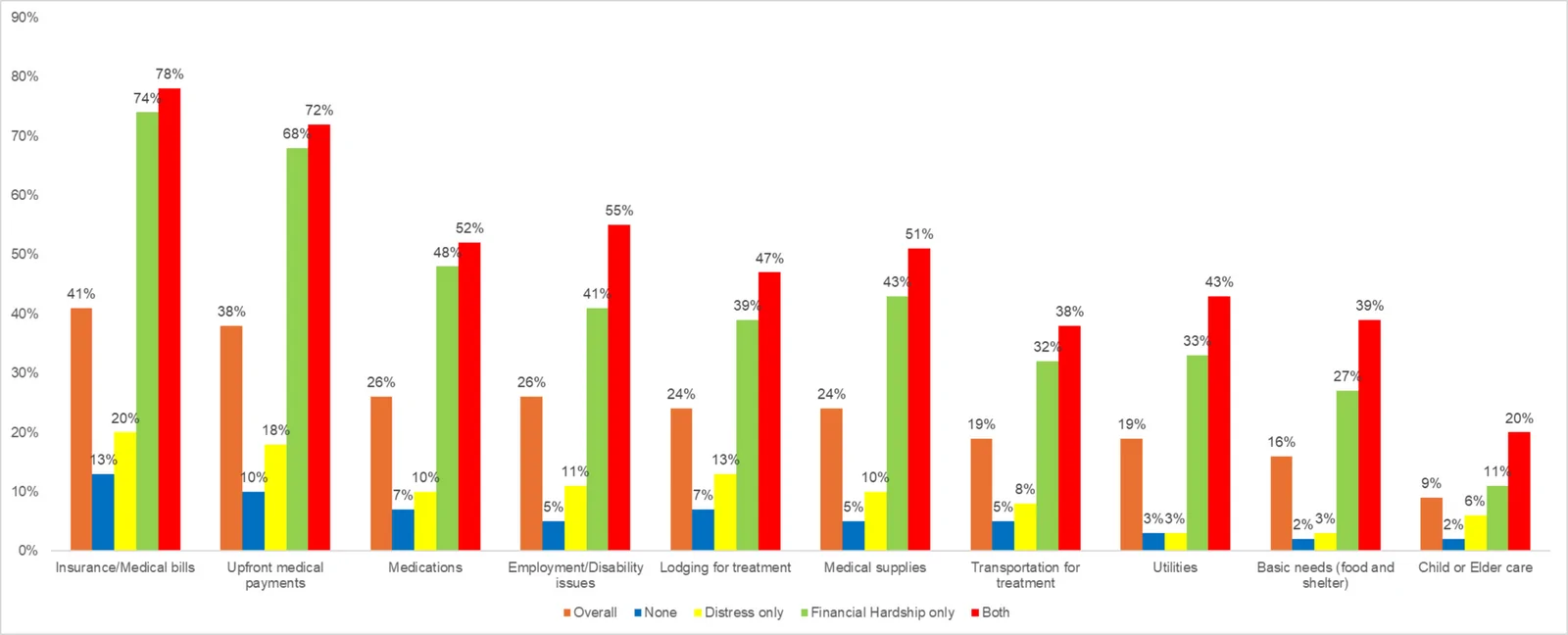
Domains of reported financial difficulty overall and by distress (*N* = 4637)

Overall, the most frequently endorsed financial difficulties within our sample were insurance or medical bills (41%), upfront medical payments (38%), employment or disability issues (26%), and medications (26%; Fig. 1). Compared to patients reporting neither financial hardship nor distress or distress only, patients reporting financial hardship only or co-occurrence more often reported financial difficulties across each of the domains, with more than two-thirds reporting difficulties with insurance, medical bills, or upfront medical payments, and about half reporting difficulties affording medications. Compared to those with financial hardship only, patients with co-occurring financial hardship and distress more often reported each of the financial difficulties, with the largest differences seen in problems with employment and disability issues and affording utilities and basic needs.

### Factors associated with financial hardship and distress

Compared with those reporting neither financial hardship as measured by the COST instrument nor distress, patients reporting both conditions were generally younger (median age 58 [IQR 48–67] vs 69 [IQR 60–76]), less educated (30% with high school education or less vs. 14%), not married (49% vs. 35%), non-White (29% vs 20%), or female (69% vs 61%; Table 1). Results were similar in adjusted models (Table 3). Lower educational attainment and patients unmarried demonstrated the strongest associations with co-occurring financial hardship and distress. Compared to patients holding a bachelor’s degree or higher, those with a high school education or less had 2.7-times higher odds (95% CI 2.15–3.45) and those with some college education had 2-times higher odds (95% CI 1.60–2.52) of reporting both financial hardship and distress. Patients who were not married also had 1.8-times higher odds of experiencing both financial hardship and distress compared with married patients (95% CI 1.50–2.13). Patients who were younger, non-white, female, or living in a highly disadvantaged neighborhood also had significantly higher odds of co-occurring financial hardship and distress compared to their counterparts.

**Table 3:** Adjusted models estimating associations between patient sociodemographic and clinical characteristics and reported financial hardship and psychological distress (*N* = 4637)

	**Both vs. neither** **OR (95% CI)**	**Psychological distress vs. neither** **OR (95% CI)**	**Financial hardship vs. neither** **OR (95% CI)**
Age	**0.95 (0.94–0.95)**	**0.99 (0.99–0.99)**	**0.96 (0.95–0.97)**
Non-White vs. White	**1.39 (1.14–1.70)**	0.92 (0.74–1.13)	**1.44 (1.12–1.87)**
Females vs. males	**1.37 (1.13–1.66)**	**1.34 (1.09–1.61)**	0.90 (0.70–1.16)
Other vs. married	**1.79 (1.50–2.13)**	0.94 (0.79–1.13)	**1.68 (1.34–2.10)**
Some college vs. bachelors’/higher	**2.01 (1.60–2.52)**	1.17 (0.94–1.46)	**1.70 (1.26–2.30)**
High school education/less vs. bachelors’/higher	**2.73 (2.15–3.45)**	1.18 (0.94–1.50)	**2.04 (1.50–2.79)**
Most vs. least neighborhood disadvantage	**1.34 (1.07–1.67)**	1.02 (0.80–1.29)	**1.42 (1.08–1.88)**
Rural vs. urban residence	1.16 (0.93–1.45)	0.91 (0.72–1.14)	1.03 (0.77–1.38)
Gastrointestinal vs. breast	1.06 (0.77–1.47)	**0.58 (0.42–0.81)**	0.78 (0.50–1.20)
Gynecological vs. breast	0.73 (0.52–1.03)	0.74 (0.54–1.02)	0.75 (0.48–1.18)
Other vs. breast	0.96 (0.75–1.22)	**0.76 (0.60–0.96)**	0.92 (0.67–1.27)
Advanced vs. early	1.07 (0.83–1.38)	0.80 (0.63–1.03)	**1.43 (1.03–1.98)**

Bolded estimates indicate *p* <.05

Patients with higher odds of reporting distress only were female compared to male (aOR 1.34, 95% CI 1.09–1.61), while patients with lower odds of reporting distress only were younger (aOR 0.99, 95% CI 0.99–0.99 for 1-year increase in age). Associations for patients reporting only financial hardship were similar to the co-occurring group with one exception. Patients with advanced stage cancer had 1.4 times higher odds of reporting financial hardship only compared to those with early stage cancer (95% CI 1.03–1.98).

## Discussion

This analysis of standard-of-care collected, ePRO survey data from patients initiating medical oncology treatment at a large academic medical center in Alabama found both psychological distress and financial hardship were common and co-occurring, with almost one-third of patients experiencing both financial hardship and distress. Furthermore, patients experiencing financial hardship had almost three times the odds of also experiencing psychological distress compared to those without financial hardship, and financial hardship and distress escalated concurrently. These results, which support findings from previous studies [10, 27], demonstrate a substantial burden for patients with cancer and underscore the fact that the co-occurrence of financial hardship and psychological distress cannot be ignored. Though our institution has implemented screening for both financial hardship and distress, interventions to address the psychological impact of financial hardship are needed due to its impact on emotional well-being and quality of life. Evidence-based interventions such as oncology financial navigation have demonstrated effectiveness in reducing the material and behavioral consequences of financial hardship [28–34], yet few interventions have been shown to be effective in addressing the psychological impact of financial hardship [35, 36], including distress. Thus, pairing financial navigation with evidence-based interventions to address distress, such as psycho-oncology, may aid in addressing the psychological impact of financial hardship. As part of standard care, UAB offers psycho-oncology services to address psychological, social, and behavioral aspects of cancer and improve emotional and mental well-being throughout the cancer experience [37]. Psycho-oncologists help patients cope with emotional distress and offer supportive care (e.g., psychotherapy, medications, and strategies for coping with pain, insomnia, and other symptoms) [38]. Provision of evidence-based psycho-oncology care has been shown effective in reducing depression and anxiety, alleviating distress, and fostering resilience [39, 40]. Future studies should investigate how this standard-of-care delivered supportive care service, offered both alone or coupled with financial navigation, can become a potential avenue for tailoring to more specifically address the psychological effects of financial hardship.

In our study, factors associated with reporting both financial hardship and psychological distress included patients who were younger, less educated, unmarried, female, non-White, and resided in highly disadvantaged neighborhoods. These results emphasize the fact that financial hardship and psychological distress do not occur in isolation, but reflect a complex web of cumulative disadvantage across social, economic, and political systems. As education level showed the strongest association with co-occurring financial hardship and distress, interventions to address financial or health insurance literacy may be an appropriate and impactful pathway to reduce both financial hardship and distress. For example, Park and colleagues are currently testing the Health Insurance literacy Navigation Tools (HINT) intervention for cancer survivors, which has decreased patient psychological financial hardship in pilot testing [41]. Conversely, women in our study were significantly more likely than men to report psychological distress unrelated to financial hardship. This distress may stem from a variety of factors. Women often carry the majority of caregiving or family management responsibilities, which could be challenging to maintain when going through active cancer treatment [42]. Women also are more likely to be diagnosed with cancer that affect their sexuality and fertility, potentially altering their sense of identity or body image [43]. Furthermore, depending on their cancer type, women can receive hormone-based cancer treatments with psychological side effects, including depression and insomnia [44, 45]. Interventions such as the Promoting Resilience In Stress Management (PRISM) program by Rocque and colleagues may target and address distress-related issues specifically for women with cancer, as pilot results have shown increases in patient resilience, growth, and self-improvement [46]. Lastly, patients in our study diagnosed with an advanced cancer were significantly more likely than those with an early-stage cancer to report financial hardship unrelated to distress. This financial hardship may stem from patients with recurrent cancer, who have experienced a prolonged treatment course, which may result in high out-of-pocket expenses, depletion of financial reserves, reaching insurance coverage benefit limits, and large gaps in employment [47, 48]. Furthermore, these patients may be less distressed due to their knowledge about what to expect regarding treatment course and familiarity with the healthcare system. Financial hardship screening interventions have shown promise in reducing the development or worsening of financial hardship for patients with metastatic cancer, such as those tested within a large, multi-site randomized controlled trial by Blinder and colleagues [49]. Importantly, this screening also increased connections to financial assistance to address identified financial hardship. Future studies should consider the implications of these findings, which showed differential patterns of financial hardship and distress by patient-level factors, and potential interventions when identifying the supportive services that may be most appropriate across different patient populations.

Patients in our study with co-occurring financial hardship and distress or financial hardship only were similar in their reporting of financial difficulties. However, patients with both financial hardship and distress most often reported problems with employment and disability issues, as well as difficulty affording utilities and basic needs. These results are unsurprising, as employment disruptions, which are directly linked to reductions in earned income and loss of health insurance, as well as difficulty affording everyday needs essential to survival, are inherently distressing [50, 51]. To appropriately respond to screen-detected employment issues and thus reduce financial hardship and distress, it is imperative to ensure a healthcare team member is trained in the legal complexities of employment, including Title I of the Americans with Disabilities Act, the Family and Medical Leave Act, state-specific paid and unpaid leave laws, disability insurance, and Social Security Disability benefits. Non-profit organizations, such as the American Cancer Society and CancerCare, as well as local organizations often provide small grants or assistance with paying for basic needs for patients going through active cancer treatment. Future efforts should seek to understand which resources aid in best reducing cancer-related financial hardship and distress.

Our study provides an examination of the relationship between financial hardship and psychological distress for patients with cancer in Alabama, a state characterized by high poverty rates, large rural populations, and reduced access to oncologic and psychologic care. Our study examined data for patients who had access to resources of a large, academic medical center. Other Alabamians with cancer may not have access to the same resources and may experience greater financial hardship and psychological distress. This sample bias is important to note, as UAB has the available workforce, information technology infrastructure, and financial capacity to implement standard-of-care distress and financial hardship screening for their medical oncology patients [10, 11, 17]. This is due, in part, to their participation in the Center for Medicare and Medicaid Innovation’s Oncology Care Model and its successor, the Enhancing Oncology Care Model [52, 53]. These value-based payment models incentivized participating oncology practices to improve care coordination, quality, and cost-efficiency for patients on active systemic treatment. CMS has also recently implemented principal illness navigation billing codes to reimburse practices for health-related social needs navigation, including financial navigation. This is a policy-level solution which may aid in the provision of financial hardship and distress screening and referrals within clinical practice, as well as tracking this data at the system-level to understand patient need [54]. However, oncology practices in rural or community-based settings may not have the capacity to implement these supportive care screenings, nor have resources available for appropriate referrals to financial, social work, or psycho-oncology services based on positive screens. Thus, community-driven approaches, such as community health advisor based models in partnerships with academic cancer centers [55–57], or digital health options [58, 59] may be promising opportunities to reduce cancer-related financial hardship and psychological distress experienced by Alabamians with cancer.

This study should be considered within the context of limitations. This study was a secondary analysis of standard-of-care screening items, and may suffer from residual confounding due to the inability to collect data for research purposes. Data for this study was from a single-item distress scale. However, this scale is the recommended screening tool for distress. Patients completed these screening items at the initiation of cancer treatment. Thus, our study does not address longitudinal distress and financial hardship experienced by patients throughout cancer treatment. However, we expect to explore this in future work.

## Conclusion

In this study of patients initiating medical oncology treatment at a large, academic medical center in Alabama, one-third of patients reported co-occurring financial hardship and psychological distress. Furthermore, our study found financial hardship and distress escalated concurrently. Our findings support integration of routine financial hardship and psychological distress screening, with referrals to tailored interdisciplinary interventions such as psycho-oncology services and interventions addressing employment issues and affordability of basic needs, to address both the financial and psychological needs of patients with cancer.

## Supplementary Information

Below is the link to the electronic supplementary material.

ESM1 (DOCX 14.5 KB)

## Data Availability

The data that support the findings of this study are available on request from the corresponding author. The data are not publicly available due to privacy or ethical restrictions.
