# Supplementary material for: Associations between psychological distress and financial hardship for patients with cancer receiving care in Alabama
Source: J Cancer Surviv. 2026 Apr 1;20(Suppl 2):1779–88. doi: 10.1007/s11764-026-02012-6 (PMC13744508; doi:10.1007/s11764-026-02012-6)
Supplement: Supplementary file 1 [file 11764_2026_2012_MOESM1_ESM.docx]

Supplemental Table 1. Comparison of patients included vs. excluded (N=9,799).

|  | **Included**  **(N=4637)** | **Excluded**  **(N=5162)** |
| --- | --- | --- |
|  | **n (%)** | **n (%)** |
| **Age, Median (IQR)** | 65 (53 – 73) | 66 (53 - 75) |
| **Sex** |  |  |
| Female | 3037 (65) | 3459 (67) |
| Male | 1600 (35) | 1703 (33) |
| **Race** |  |  |
| White | 3163 (68) | 3563 (69) |
| Non-White^a^ | 1080 (23) | 1032 (20) |
| Unknown | 394 (9) | 567 (11) |
| **Marital Status** |  |  |
| Married | 2598 (56) | 2788 (54) |
| Other^b^ | 1846 (40) | 2219 (43) |
| Unknown | 193 (4) | 155 (3) |
| **Education** |  |  |
| High School Education/Less | 998 (22) | 1187 (23) |
| Some College^c^ | 1033 (22) | 1032 (20) |
| Bachelors’/Higher | 1409 (30) | 1704 (33) |
| Unknown | 1197 (26) | 1239 (24) |
| **Neighborhood disadvantage** |  |  |
| Least Disadvantaged | 2600 (56) | 2994 (58) |
| Most Disadvantaged^d^ | 884 (19) | 723 (14) |
| Unknown | 1153 (25) | 1445 (28) |
| **Residence** |  |  |
| Rural | 773 (17) | 981 (19) |
| Urban | 2744 (59) | 2994 (58) |
| Unknown | 1120 (24) | 1187 (23) |
| **Cancer Type** |  |  |
| Breast | 1076 (23) | 1291 (25) |
| Gastro-intestinal | 466 (10) | 413 (8) |
| Gynecological | 397 (9) | 464 (9) |
| Other | 2175 (47) | 2323 (45) |
| Unknown | 523 (11) | 671 (13) |
| **Cancer Stage** |  |  |
| Early | 1060 (23) | 1394 (27) |
| Advanced^e^ | 982 (21) | 1239 (24) |
| Unknown | 2595 (56) | 2529 (49) |

^a^Black/African American, Hispanic/Latinx, Asian, American Indian/Alaskan Native, Multiple races; ^b^Divorced, separated, widowed, single; ^c^Associate’s or junior college degree; ^d^Area Deprivation Index percentile of 85-100; ^e^Stage III/IV
